# 5-Fluorouracil and Curcumin Combination Coated with Pectin and Its Strategy towards Titanium Dioxide, Dimethylhydrazine Colorectal Cancer Model with the Evaluation of the Blood Parameters

**DOI:** 10.3390/polym14142868

**Published:** 2022-07-14

**Authors:** Chenmala Karthika, Md. Habibur Rahman, Raman Sureshkumar, Rokeya Akter, Azmat Ali Khan, Amer M. Alanazi, Abul Kalam Azad, Paritosh Barai, Hasi Rani Barai

**Affiliations:** 1 Department of Pharmaceutics, JSS College of Pharmacy, JSS Academy of Higher Education and Research, Ooty 643001, India; karthika1994haridas@gmail.com; 2 Department of Global Medical Science, Wonju College of Medicine, Yonsei University, Wonju 26426, Korea; pharmacisthabib@gmail.com (M.H.R.); rokeyahabib94@gmail.com (R.A.); 3 Pharmaceutical Biotechnology Laboratory, Department of Pharmaceutical Chemistry, College of Pharmacy, King Saud University, Riyadh 11451, Saudi Arabia; azkhan@ksu.edu.sa (A.A.K.); amalanazi@ksu.edu.sa (A.M.A.); 4 Faculty of Pharmacy, MAHSA, Bandar Saujana Putra, Jenjarom 42610, Selangor, Malaysia; aphdukm@gmail.com; 5 Department of Biochemistry and Molecular Biology, Primeasia University, Dhaka 1213, Bangladesh; paritoshbarai9@gmail.com; 6 School of Mechanical and IT Engineering, Yeungnam University, Gyeongsan 38541, Korea

**Keywords:** colorectal cancer, titanium dioxide nanoparticles, dimethylhydrazine, 5-fluorouracil, curcumin, pectin

## Abstract

Colorectal cancer is considered the third most common cancer and the second leading cause of death globally. It has been proven that titanium dioxide nanoparticles produce oxidative stress and can lead to chronic inflammation, which could turn into diseases like cancer, cardiovascular disorders, diabetes, and so on. To evaluate the effect of 5-fluorouracil (5-FU) curcumin (CUR) conjugate coated with pectin on colorectal cancer induced by titanium dioxide nanoparticles (TiO_2_-NPs) and dimethylhydrazine (DMH), male rats were administered TiO_2_-NPs (5 mg/kg) orally and DMH (1 mg/kg) peritoneally for 70 days and treated with 5-FU (60 mg/kg) and CUR (240 mg/kg) conjugate (1:4 ratio) coated with pectin. The bodyweight of the animals was evaluated, and the blood sugar level was calculated. Further blood and plasma analyses were conducted. Hematological parameters, antioxidant parameters, and biochemical estimation were taken into consideration. The TiO_2_-NPs level in the blood and colorectal region was also calculated. With the induction of colon cancer using TiO_2_-NPs and DMH, a significant increase in the body weight of the animals was seen; eventually, with treatment, it was reduced. The bodyweight increase was due to an increase in the blood sugar level. There were also significant changes in the hematological parameters and biochemical estimation reports when comparing those of the positive control, negative control, and treated groups. No significant effect on biochemical estimation reports was seen. Conclusions: These reports suggest that 5-FU CUR conjugate coated with pectin helps in the management of colorectal cancer induced by TiO_2_-NPs and DMH.

## 1. Introduction

Colorectal cancer is considered the third most common cancer and the second leading source of death globally [1]. The occurrence of colorectal cancer is increasing in developed countries, as well as in middle- and low-income countries due to westernization. Unhealthy living habits, especially adaptation to fast food, packed food items, and lack of exercise, are the leading causes of the increase in the incidence of colorectal cancer [2].

Titanium dioxide nanoparticles (TiO_2_-NPs), a white-colored food additive (E171), are proven to pose a major risk to health. TiO_2_-NPs can induce inflammation due to oxidative stress and can significantly cause colorectal cancer in human beings and animals [3]. TiO_2_-NPs have a broad range of applications in the food industry, pharmaceuticals, cosmetic industry, and toothpaste. 

This idea is also used to induce colorectal cancer in the animal model. The use of TiO_2_-NPs has already been shown to cause cancer and persistent intestinal irritation [4]. Food-grade TiO_2_-NPs are frequently utilized as a white pigment in the food sector, particularly in confectionery. In carcinogenic-chemical-induced models, 100-day treatment with TiO_2_-NPs causes colon microinflammation, pre-neoplastic lesions, and the formation of aberrant crypt foci, according to research. These findings yield a risk assessment of colon cancer susceptibility in human candidates exposed to TiO_2_ through food [5]. Previous research indicates that oral treatment of TiO_2_-NPs in mice raises plasma glucose levels, and, as a result, the body weight of the animals eventually increases. As a result, oral TiO_2_-NPs and peritoneal DMH administration were chosen for colorectal cancer induction [6].

Curcumin (CUR), also known as diferuloylmethane, is a yellow pigment found in turmeric, a spice and culinary coloring. According to toxicological research, CUR is non-toxic even at large doses [7]. This drug was recently discovered to have a considerable inhibitory impact on the growth of several mouse tumors, including skin, stomach, colon, and liver malignancies. In humans, CUR is thought to be a potentially safe and non-toxic colorectal cancer chemopreventive medication [8]. As a result, CUR has already been studied as a chemopreventive drug in several preclinical investigations, and its effectiveness implies that CUR’s usage will certainly rise in the future. Because of its extensive use and multiple advantages as a chemopreventive agent in colon cancer, we believe that CUR-induced inhibition of COX-2 expression may improve the efficacy of standard anti-colon-cancer medications such as 5-fluorouracil (5-FU) [9].

5-FU is the first line chemotherapeutic agent used for the treatment of CRC in both palliative and adjuvant situations. Over the last four decades, various techniques have been developed and used to increase the anti-tumor [10] effectiveness of 5-FU and overcome clinical resistance, including the use of 5-FU-based combination regimens and 5-FU pro-drugs [11]. More than 80% of 5-FU is catabolized by hepatic dihydropyrimidine dehydrogenase (DPD), while the remaining causes cell death by blocking RNA and DNA synthesis via fluorodeoxyuridine monophosphate (FdUMP) and fluouridine triphosphate (FUTP) [12]. The most serious side effects of 5-FU include cardiotoxicity, diarrhea, dermatitis, myelosuppression, and mucositis, and it also possesses multidrug resistance (MDR) [13]. The combination of 5-FU and CUR may have a synergistic effect in the treatment of colorectal cancer. Despite recent encouraging developments in colorectal cancer therapy, patient response rates continue to be modest, and the benefit of 5-FU-based therapy is usually jeopardized by chemoresistance. Each person’s genetic and epigenetic composition may have a role in inter-individual variation in therapy response in colorectal cancer patients [14]. Dietary pectin supplementation has been demonstrated to significantly prevent colon cancer and have an anti-proliferative effect in mice’s distal colon [15]. Pectin may help prevent colon cancer by inhibiting galectin-3’s biological effects. In turn, it helps in the site-specific delivery of the conjugate to the colorectal region [16].

Here in this paper, we induce colorectal cancer in rats using TiO_2_-NPs as well as dimethylhydrazine (DMH). Then, we treat the animals using 5-fluorouracil (5-FU) and curcumin (CUR) in the ratio of 1:4. The effect of treatment is compared to that of the normal group (positive control-healthy animals) as well as colorectal cancer-induced group (negative control). Further blood parameters are analyzed, and we report on their effects.

## 2. Materials and Methods

### 2.1. Chemicals

Pectin was obtained from Sigma-Aldrich Pvt. Ltd. (Steinheim, Germany), and 5-FU and DMH from Avra Synthesis Pvt. Ltd. (Hyderabad, India). TiO_2_ was obtained as a gift sample from Bimal Pharma Pvt. Ltd. (Mumbai, India), and CUR was obtained as a gift sample from Himalaya Pvt. Ltd. (Bangalore, India). Before the start of the trial, all samples were tested for purity and confirmation.

### 2.2. Cell Culture

Human colorectal carcinoma cell line (HCT 116) cells were obtained from the American Type Culture Collection and was cultured at 37 °C in a humidified atmosphere containing 5% CO_2_ in RPMI-1640 (Invitrogen, Carlsbad, CA, USA) supplemented with 10% FBS and a 1% antibiotic-antimycotic cocktail. In tissue culture flasks, the cells were trypsinized using a PBS solution containing 0.25% trypsin and 0.03% EDTA until they achieved 70% confluence.

#### Cell Cytotoxicity Studies

To measure cell cytotoxicity, an MTT reduction assay was used. HCT-116 cells were seeded into a 96-well plate (5 × 10^3^ cells per well) and incubated for 24 h at 60–70% confluence under conventional growth conditions. Cells were subsequently treated for 48 and 72 h with formulations of 5-FU, CUR, 5-FU+CUR (1:4 ratio) coated with pectin, and 5-FU+CUR (1:4 ratio). Following the drug treatment, the cells were incubated for 4 h with 10% *v*/*v* MTT (5 mg mL^−1^ dissolved in PBS pH 7.4). The medium was then entirely removed, and the resulting formazan crystals were dissolved in 100 µL isopropyl alcohol. A Biorad plate reader Model 680 was used to detect absorbance at 570 nm [6]. 

### 2.3. Pectin Coating

To achieve total solubility, pectin powder (6% *w*/*v*) was dissolved in distilled water at 20–22 °C and agitated at 500 rpm for 30 min. To coat, 10 mL of pectin solution was combined with 1 g of conjugated powder and spun for 15 min at 500 rpm. A physical conjugate of 5-FU:CUR (1:4 ratio; 60 mg of 5-FU and 240 mg CUR) was coated with pectin. The pH of the conjugates was also noted [17].

### 2.4. Colorectal Cancer Induction in Animals

For a total of 70 days, the Sprague–Dawley male rats were given 5 mg/kg body weight TiO_2_-NPs (oral route) 5 days per week and 1 mg/kg body weight DMH (peritoneal route) 1 day per week. Body mass index and hematological findings were obtained. The animal’s weights were recorded every week.

#### Therapy

Following the conclusion of the 70-day period, the treatment was started. The first group serves as a positive control, while the second serves as a negative control (induction of CRC without therapy), with both groups receiving only ordinary saline. The third group received treatment with 60 mg of 5-FU and 240 mg of CUR (1:4) coated with pectin, whereas the fourth group received negative control therapy with 60 mg of 5-FU and 240 mg of CUR (1:4) coated with pectin. Intraperitoneally, the pectin-coated conjugate (a physical conjugation of 5-FU and CUR) was dissolved in phosphate buffer saline (PBS), whereas the control group, group 1, received phosphate buffer saline (PBS) solution. The Sprague–Dawley rats were kept in a room with a 24 h dark cycle, temperature of 24 ± 2 °C, and relative humidity of 60 ± 5%. All rats’ weight and diarrhea scores were recorded daily at the beginning the day after the treatment and induction [6].

### 2.5. Analyze Your Body Weight

Bodyweight measurements were obtained once a week. The animals’ body weights were assessed before and after the cancer induction (on Sunday). Before and after induction, as well as during and after therapy, the animals were weighed. Following that, the animals were given unrestricted access to water, and their fasting blood glucose levels were tested [18].

### 2.6. Elemental TiO_2_-NPs Concentrations in the Blood Plasma and Colon

0.1–0.5 g samples of blood plasma and colon were treated with 10 mL of nitric acid and 2 mL of perchloric acid in a 100 mL glass beaker, respectively. After that, the samples were digested for one hour at 230 °C. The digestion solution was heated to 280 °C until nearly completely evaporated, then cooled to room temperature and diluted with ultrapure water to a final volume of 25 mL. The concentrations of TiO_2_-NPs were determined using an inductively coupled plasma-mass spectrometer (ICP-MS, Varian 820-MS, Palo Alto, CA, USA) [4].

### 2.7. Hematological Parameters

At the time of sacrifice, all the animals were subjected to blood collection to measure hematological parameters. A retro-orbital puncture was used to collect blood. The above parameters were studied to learn more about the effects of CUR and 5-FU individually and in combination on the following specific blood components:

- RBC;
- WBC;
- Hemoglobin-Hb content;
- Platelet count.

Following the retro-orbital puncture, blood was collected into pre-coated (Heparin 25 IU/mL) blood collection tubes. Using a cell counter, the concentrations of these different components were then determined [19].

### 2.8. Antioxidant Studies

For estimation of the molecular anticancer potential, antioxidant activities were evaluated [20].

#### 2.8.1. Determination of Nitric Oxide (NO)

We started with 20 µL organ + 80 µL of PBS. Then, after 5 min, we added the 50 µL of salphanilamide solution (0.082 g + 25 mL distilled water). Then, we added 50 µL of NED solution (0.025 g + 25 mL distilled water) and waited for 10 min before measuring absorbance at 405 nm against a blank solution [21].

#### 2.8.2. Determination of Advanced Protein Oxidation Products (APOP) Assay

We started with 10 µL organ + 190 µL of PBS. Then, we added 100 µL of acetic acid and 100 µL of KI (0.0134 g + 3 mL distilled water) and waited 2 min. Later, absorbance at 405 nm was measured in comparison to a blank solution [22].

#### 2.8.3. Detection of the Reactive Oxygen Species (ROS) Level

The levels of ROS were determined using dichloro-dihydro-fluorescein diacetate (DCFH-DA) reagent (Sigma, St. Louis, MO, USA) according to the manufacturer’s instructions. In a 96-well black plate, 10 µL of serum and brain lysate samples were combined with 100 µL of 10 µM DCFH-DA and incubated for 30 min at 37 °C. The fluorescence was measured at 488 nm excitation or 525 nm emission using a DTX multi-mode microplate reader (Beckman Coulter Inc., Brea, CA, USA) [23]. 

#### 2.8.4. Catalase (CAT)

Hydrogen peroxide (H_2_O_2_) is said to be an oxidative stress inducer. In turn, it gets broken down into peroxidase free radicals (O_2_^−^) and water (H_2_O). Oxidative stress that aids in carcinogenesis is induced by O_2_^−^ free radicals. Hence, the potential of catalase to oxidase H_2_O_2_ was evaluated. Colonic mucosa homogenate (100 µL) was added into potassium phosphate buffer (2.25 mL) and was then incubated for 30 min at 25 °C. This was followed by the addition of 650 µL of 7.5 mM H_2_O_2_ into the mixture. The alteration in absorbance per minute for 2–3 min was analyzed using UV–visible spectrometry at 240 nm and was reported as µmol/min/mg of protein [24]. 

#### 2.8.5. Superoxide Dismutase (SOD)

For the assay, the method demonstrated by Winter Bourn et al. was adapted. Around 100 µL of the mucosa and homogenate supernatant was added into 8.3 pH 0.052 M sodium phosphate buffer (1.2 mL) with 186 µM phenazine methosulphate (100 µL) and nitro blue tetra tetrazolium (300 µL). In addition, 750 µM NADH (200 µL) was added and incubated for 90 s at 30 °C. To halt the reaction of the mixture, 100 µL of glacial acetic acid was added, followed by the addition of N-butanol (4 mL), and the mixture was then sonicated continuously. The chromogen intensity in butanol was evaluated by UV–visible spectrometry at 560 nm. The SOD content was expressed in U/mg of tissue [25].

The estimation of glutathione was also performed as per the method demonstrated earlier. The tissue and mucosa of the colon were homogenized along with 0.2 M ice-cold perchloric acid, and 0.01% EDTA. This was followed by its centrifugation at 4 °C and 10,000 rpm for 10 min. Then, 0.3 mM of reduced NADPH (500 µL), 6 mM DTNB, and 25 units/µL of glutathione reductase (10 µL) freshly prepared in pH 7.5 phosphate buffer were added. Initiation of the reaction was carried out with the addition of 200 µL homogenate supernatant and the reaction mixture into a cuvette. The absorbance was measured at a 412 nm wavelength at 30 °C, and the amount of glutathione was analyzed every 3 min. The change in the test solution absorbance was compared with that of the standard and was indicated as nmol/mg protein [26].

#### 2.8.6. Malondialdehyde

The thiobarbituric acid reactive substances (TBARS) level in colonic mucosa was estimated by using the standard method: lipid peroxidation marker with the addition of 10% mucosal homogenate (0.4 mL), 8.1% sodium dodecyl sulfate (1.5 mL, 3.5 pH), 20% acetate buffer (1.5 mL), and 0.8% solution of thiobarbituric acid (1.5 mL) were added, sonicated, and heated for 1 h at 95 °C. The mixture was then cooled, and a 15:1 ratio of n-butanol-pyridine (5.0 mL) was added. The n-butyl-pyridine layer absorbance was analyzed at 532 nm. The levels were evaluated as mM/100 g of tissue [27]. 

### 2.9. Biochemical Estimation 

#### 2.9.1. Liver Enzymes Analysis

To evaluate whether the drug conjugate has any effect on nearby tissues, liver enzyme analysis was performed and estimated. The two major enzymes involved are alanine transferase (ALT) as well as aspartate transferase (AST). An estimation kit procedure was performed for the analysis. The analysis was performed on mucosal homogenate, and liver tissue was centrifuged for 10 min at 400 rpm for separation of the supernatant. A multimode microplate reader was used for the estimation of the respective wavelengths of ALT and AST [28].

#### 2.9.2. Kidney Function Analysis

Toxic activity with the adjoining kidney is analyzed using an estimation of blood urea nitrogen. The readings were analyzed using a multimode plate reader at their determined wavelength [29].

#### 2.9.3. Lowry’s Method for Protein Content Estimation

For the evaluation of total protein content, Lowry’s method was used. For obtaining the supernatant for this assay, the tissue samples were subjected to homogenization and centrifugation for 10 min at 400 rpm. Initially, 0.05–1 mg/mL of standard bovine serum albumin (BSA) was prepared. The protein sample (0.2 mL) with an analytical reagent, which was an alkaline copper sulfate solution (2 mL), was sonicated, followed by its incubation for 10 min. A Folin–Ciocalteu solution (0.2 mL) was pipetted and added to this mixture, and then incubated for around 30 min until a blue coloration was obtained. This was followed by measuring its absorbance at 660 nm [30].

### 2.10. Histopathological Report

Each colon tissue sample was submerged in a 10% formaldehyde solution before being processed for histopathology. The tissues were immersed in molten liquid paraffin before being hardened into blocks, making slicing and staining simpler. Using a rotary microtome, tis-sue-paraffin blocks were cut into 6-µm-thick slices (Leica, UK; Model No. RM2135). After being placed on staining stands, tissue slices were stained with hematoxylin and eosin. A digital microscope was used to perform pathological investigations on tissue slices. 

### 2.11. Statisticss

All datas are presented as the mean standard deviation of the mean (SEM). GraphPad Prism Software, version 9.3, was used to analyze the data, which included a one-way ANOVA followed by a Newman–Keuls post hoc test. In all situations, statistical significance was defined as *p* < 0.05 [31].

## 3. Results

### 3.1. In Vitro Study 

HCT-116 cells were used to evaluate the cytotoxic effects of 5-FU, CUR, 5-FU+CUR (1:4 ratio) coated with pectin, and 5-FU+CUR (1:4 ratio). The treatment was conducted for 48 and 72 h. A report on their comparison is depicted in Table 1. 

Evaluation and the comparison between 5-FU, CUR, 5-FU+CUR (1:4 ratio) coated with pectin, and 5-FU+CUR (1:4 ratio) showed a higher concentration in a majority of the tests. The 5-FU+CUR (1:4 ratio) at 48 h and 72 h show more cytotoxicity than pure 5-FU and CUR. The 5-FU+CUR (1:4 ratio) coated with pectin provides with a better effect than 5-FU+CUR (1:4 ratio) without pectin coating. 

### 3.2. Induction and Coating

TiO_2_-NPs and DMH were found to be effective for the induction of colorectal cancer in animals. The incidence of colorectal cancer was identified within 70 days of administration, and the mortality rate in animals was not determined. The ratio used for the physical conjugation of 5-FU and CUR was 1:4, and this conjugate was coated with pectin. The pH of the pectin-coated conjugate was found to be 6.4, and the relevant blood parameters were measured.

#### 5-FU+CUR Coated with Pectin’s Effect on TiO_2_ and DMH-Induced Rats’ Body Weight, Food, and Water Consumption

**Figure 1:**
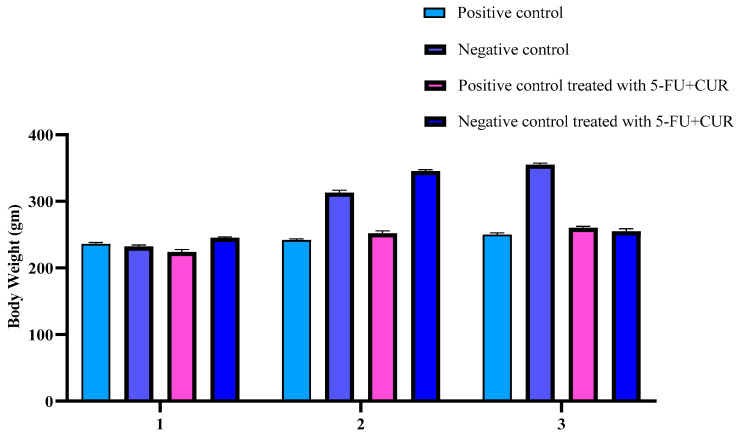
Bodyweight analysis during induction and treatment. The data are presented as the mean ± SD (*n* = 6 per group). The significance value (*p*-value) was found to be *p* < 0.05 (statistically significant).

Bodyweight, food, and water consumption are crucial indicators of the health of experimental animals. The body weight analysis of different groups was made before the treatment and after the treatment. During the induction of cancer from the initial body weight to the final body weight, there was an increase in the body weight of the animals. When it came to treatment, the body weight of the animals returned to normal. A detailed report is provided in Table 2, and graphical representation are provided in Figure 1. In this study, however, no significant differences in food intake or water intake were seen across the groups. The increase in the body weight of the animals may be due to the change in blood sugar levels.

#### 3.3. 5-FU+CUR Coated with Pectin’s Effect on TiO_2_ and DMH-Induced Rats’ Blood Sugar Level

**Figure 2:**
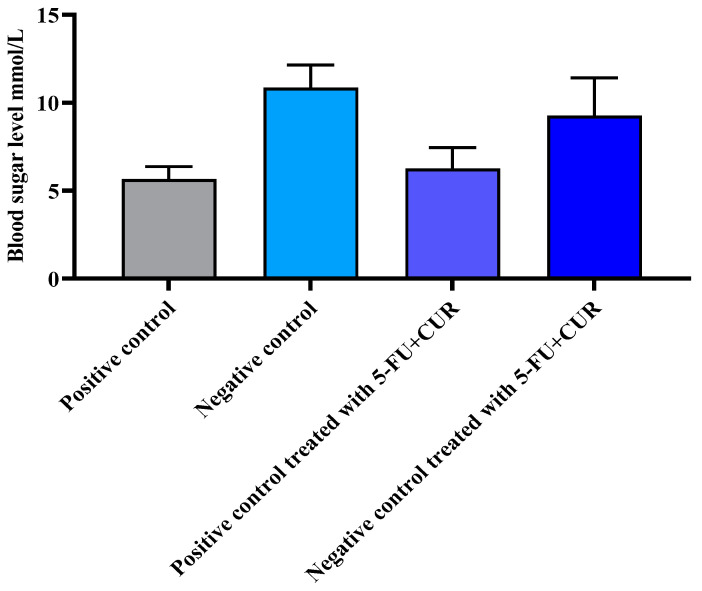
The blood sugar levels of TiO_2_-and-DMH-induced rats were affected by 5-FU+CUR coated with pectin. The data are presented as the mean ± SD (*n* = 6 per group). The significance value (*p*-value) was found to be *p* < 0.05 (statistically significant).

The blood sugar level of the animals was taken to identify the reason for the increase in weight of the animals. TiO_2_-NPs can have the ability to increase the blood sugar level of animals. The normal blood sugar of animals was found to be 5.65 ± 0.72 mmol/L. With the induction of colorectal cancer using TiO_2_ and DMH, there was found to be an increase in the blood sugar level of the animals. The relevant data and report are provided in Figure 2 and Table 3.

### 3.4. TiO_2_ Level in Blood and Colon

**Figure 3:**
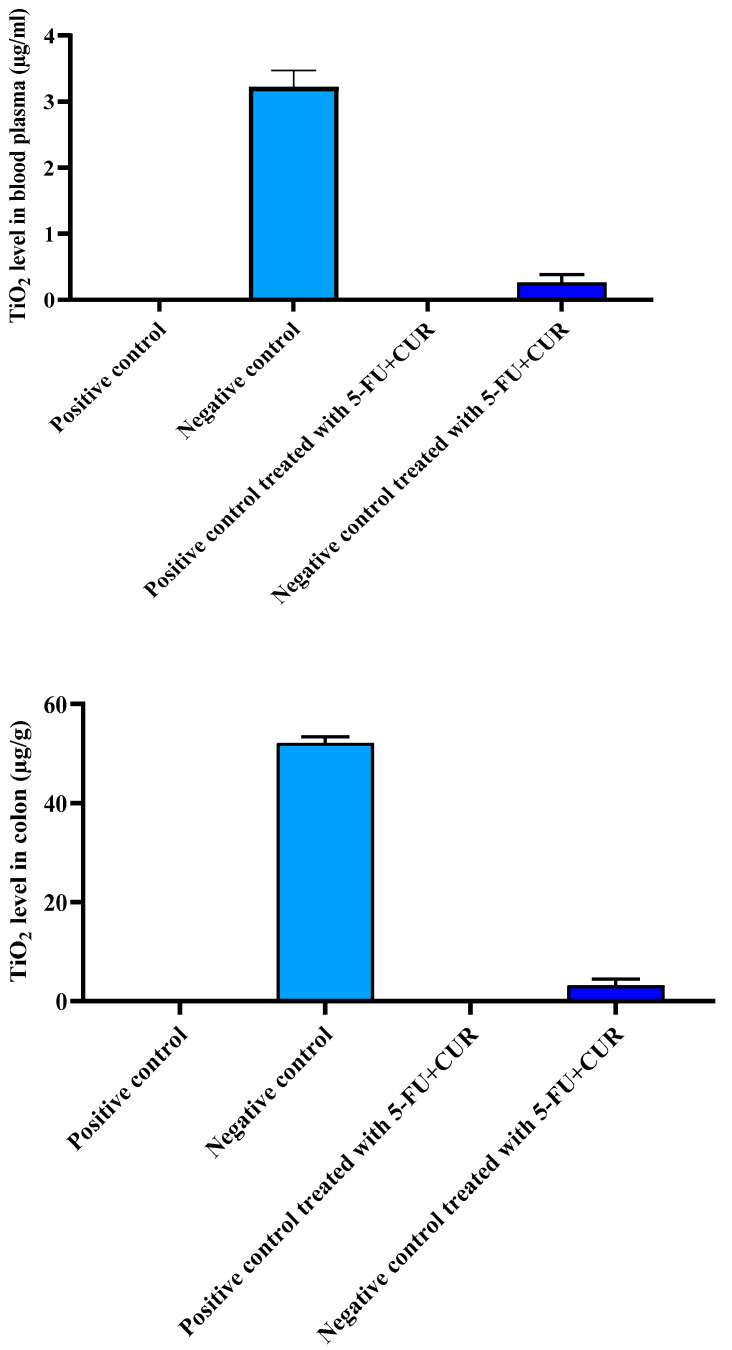
The level of TiO_2_-NPs in the blood and colorectal region. The data are presented as the mean ± SD (*n* = 6 per group). The significance value (*p*-value) was found to be *p* < 0.001 (statistically significant).

The level of TiO_2_-NPs level in the blood and the colon was conducted to evaluate the amount of TiO_2_-NPs deposition in these organs and tissues. The quantity of TiO_2_-NPs was found to be higher in the colon than that in the blood, hence colon region is the foundation to be the major target for TiO_2_-NPs deposition, which is the major reason behind the colorectal cancer occurrence in rats. The values for the content of TiO_2_-NPs in the different group was given in Figure 3 and Table 4.

### 3.5. Hematological Parameters

**Figure 4:**
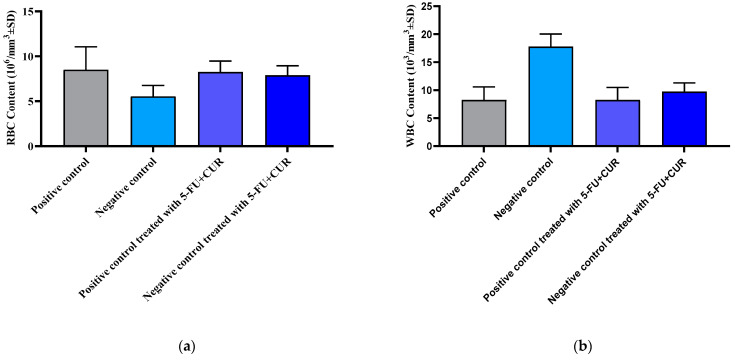

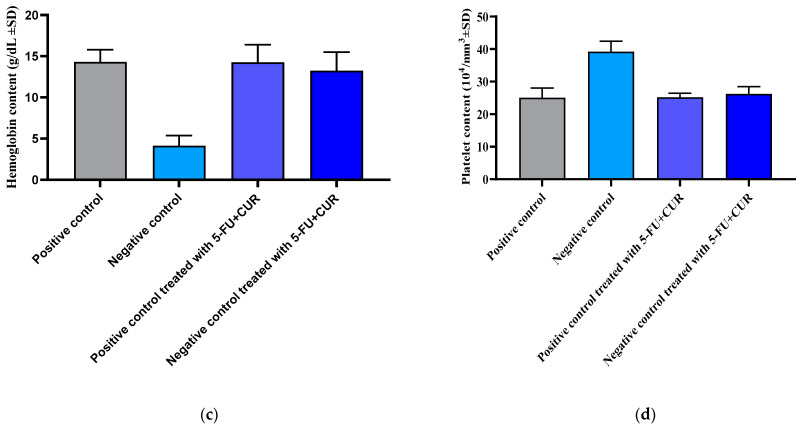
Hematological parameters of different groups. (**a**) RBC content of different groups. (**b**) WBC content of different groups. (**c**) Hemoglobin content of different groups. (**d**) Platelet content of different groups. Data were taken as mean ± SD, and *p* < 0.05 was considered to be statistically significant (*n* = 6). The significance value (*p*-value) was found to be *p* < 0.001 (statistically significant). The R^2^ Value was found to be 0.8851 (one-way ANOVA with a Bonferroni correction for multiple groups and two-tailed Student’s *t*-test for individual groups). Bartlett’s statistic correlation was found to be 6.154 with a *p*-value of 0.1044; no significant difference (*p* < 0.05) was found.

Hematological parameters were measured and noted to evaluate the changes in the concentration in blood content before and after the induction and treatment (groups 1–4). The results are given in Table 5 and Figure 4.

#### 3.5.1. Red Blood Cells Count

When compared to that of the positive control, the value of the negative control where the cancer was induced was found to be less. With the incidence of cancer, the RBC count was found to be reduced compared to that of the positive control. Subsequently, reports have shown an increase in the RBC count with treatment in the negative group. The RBC content of different groups is given in Figure 4a.

#### 3.5.2. White Blood Cells Count

When compared to the positive control, the value of the cancer-induced negative control was determined to be high. The WBC count was observed to be higher with the occurrence of cancer compared to the positive control. Following that, studies showed a decline in WBC count with therapy in the negative group. Figure 4b and Table 5 depict the WBC content of various populations. 

#### 3.5.3. Hemoglobin Count

When compared to the positive control, the cancer-induced negative control was shown to have a low value. When the cancer was present, the hemoglobin count was shown to be lower than in the positive control. Following this, tests on the negative group revealed an increase in hemoglobin count following medication. The hemoglobin content of diverse populations (Table 5) is seen in Figure 4c.

#### 3.5.4. Platelet Content

The cancer-induced negative control had a higher value when compared to the positive control. When the cancer was present, the platelet count was higher than in the control group. Then, testing on the negative group demonstrated a decrease in platelet count after medication. Figure 4d and Table 5 depict the platelet content of various populations.

### 3.6. Effect of Nitric Oxide 

Nitric oxide is a signaling molecule that plays a crucial physiological function in blood pressure control.

**Figure 5:**
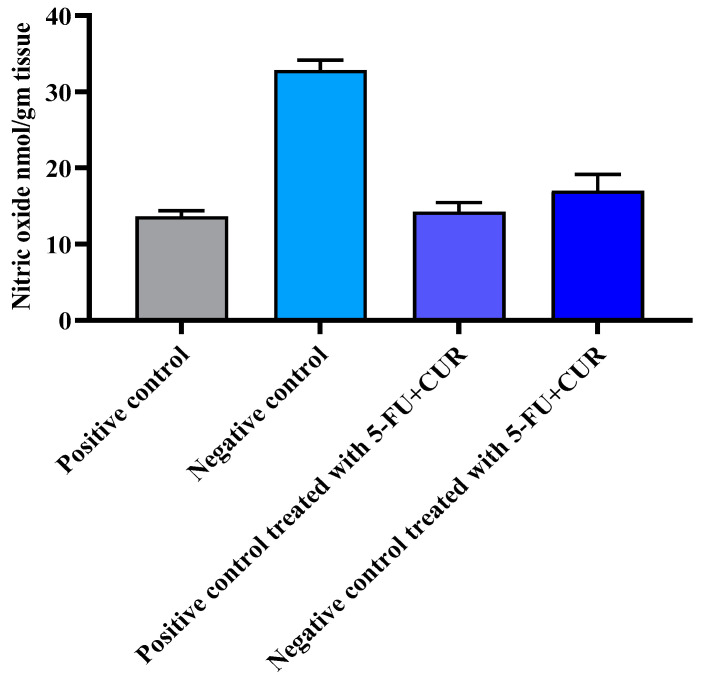
Effect of 5-FU+CUR conjugate coated with pectin on nitric oxide. The data are presented as the mean ± SD (*n* = 6 per group). The significance value (*p*-value) was found to be *p* < 0.05 (statistically significant).

However, in the presence of oxidative stress generated by the conversion of highly reactive per oxynitrate (ONOO^−^), nitric oxide is viewed in this study as an additional oxidative stress measure. As a result, a rise in nitrate levels in plasma and tissues may be linked to nitrosative stress. In this study, there is a significant increase in the plasma level of NO in TiO_2_-and-DMH-induced rats compared to the control (*p* < 0.05) (Figure 5 and Table 6). The 5-FU+CUR pectin-coated treatment restored NO levels in the TiO_2_-and-DMH-treated rats.

### 3.7. Effect on Advanced Protein Oxidation Products

**Figure 6:**
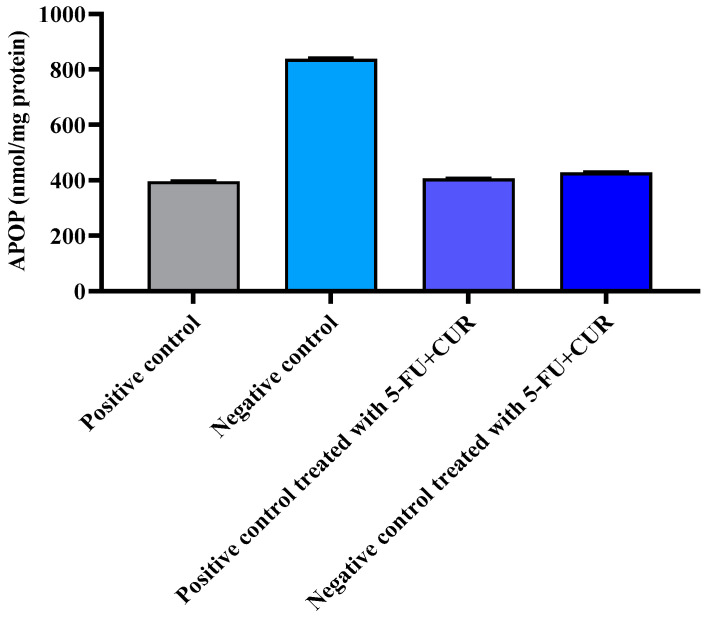
Effect of 5-FU CUR conjugate on advanced protein oxidation products (APOP). The data are presented as the mean ± SD (*n* = 6 per group). The significance value (*p*-value) was found to be *p* < 0.05 (statistically significant).

Due to oxidation, oxidative and nitrosative stress can also cause important proteins in the plasma and tissues to become inactive. As a result, tissue samples must be developed and evaluated for increased protein oxidation product (APOP). According to this study, the amount of APOP in the plasma rose significantly when compared to TiO_2_ + DMH rats. Apocynin therapy restored normal APOP levels in the plasma of TiO_2_ + DMH-treated rats (*p* < 0.05). Similarly, the noteworthy amount of APOP in liver tissue homogenates increased in the TiO_2_ + DMH treated rats, as seen in Figure 6 and Table 7. In addition, pectin-coated 5-FU+CUR therapy significantly reduced/normalized APOP levels in TiO_2_ + DMH-treated animals (*p* < 0.05).

### 3.8. Reactive Oxygen Species

**Figure 7:**
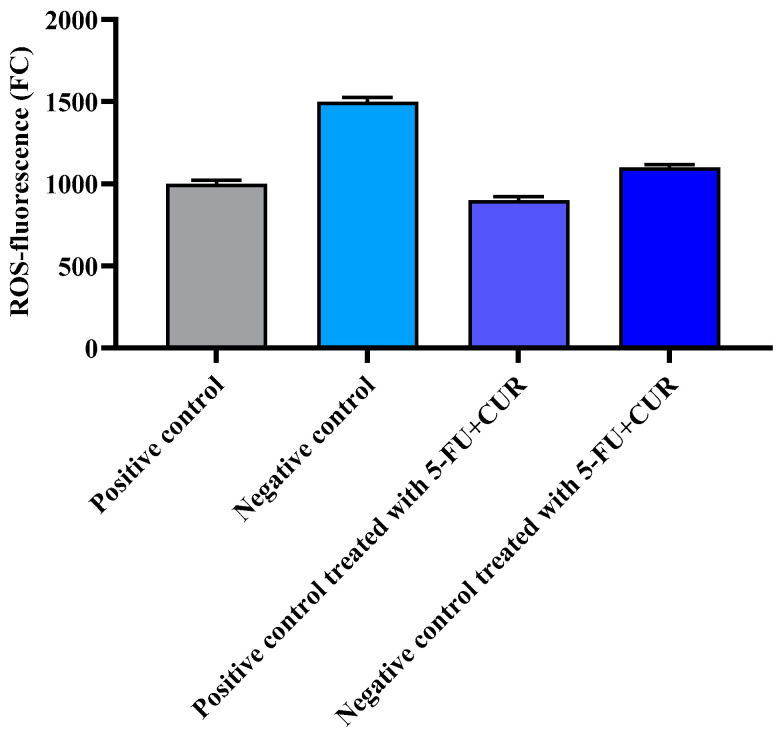
Effect of 5-FU CUR conjugate. The data are presented as the mean ± SD (*n* = 6 per group). The significance value (*p*-value) was found to be *p* < 0.05 (statistically significant).

ROS are created endogenously through several mechanisms, with the mitochondria serving as the principal source. Excessive ROS levels cause oxidative damage to DNA, proteins, and lipids, which is harmful to cells. As a result, the balance in ROS redox processes (which maintains physiological homeostasis) is critical, and its disruption affects several cellular processes associated with neoplastic transformation and abnormal growth. ROS play a variety of roles in cancer, including increased cellular proliferation, resistance to apoptosis, tissue invasion, and angiogenesis. ROS are also involved in the epithelial-to-mesenchymal transition, which is one of the most well-known metastatic routes. ROS, on the other hand, play an important role in the antitumor immune response by stimulating inflammatory and immune cells such as T lymphocytes and natural killer cells. The effect of ROS activity was found to be increased in the colon-cancer-induced group, which is the negative control group, compared to the positive control; with the treatment of the negative control, the ROS values returned to normal (Figure 7 and Table 8).

### 3.9. Antioxidant Parameters Evaluation

**Figure 8:**
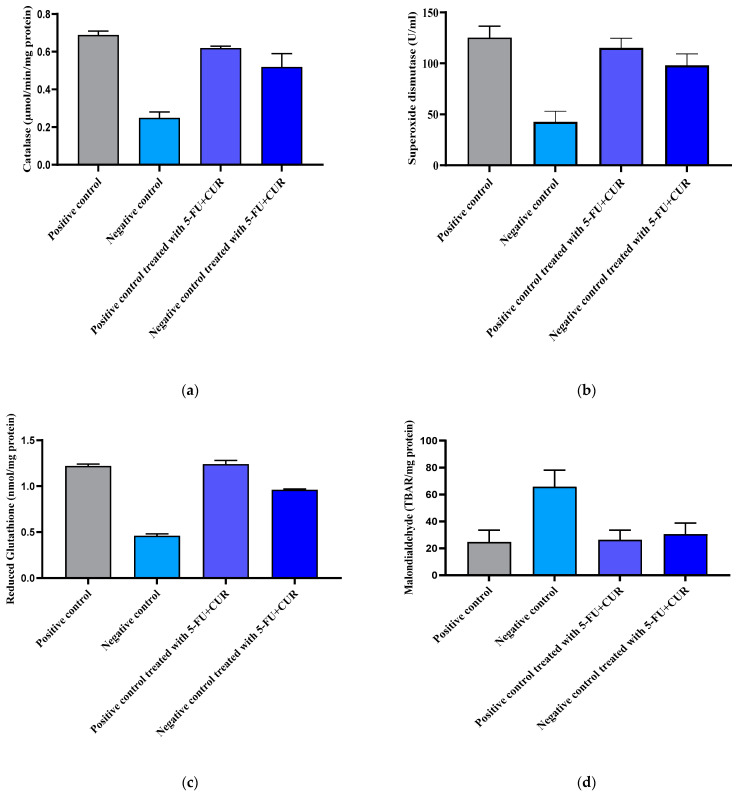
Different oxidative parameters of different groups. (**a**) Catalase activity of different groups. (**b**) Superoxide dismutase report of different groups. (**c**) Reduced glutathione activity of different groups. (**d**) Malondialdehyde activity of different groups. The data are presented as the mean ± SD (*n* = 6 per group). The significance value (*p*-value) was found to be *p* < 0.001 (statistically significant). One-way ANOVA with a Bonferroni correction for multiple groups and two-tailed Student’s *t*-test for individual groups are shown, with no significant difference (*p* < 0.05) found.

The free radical scavenging of a drug candidate demonstrates its viability in the anticancer arena. Free radicals cause the mutation and destruction of organelles and have the potential to induce uncontrolled cell differentiation. The observed results are noted and depicted in Table 9 and Figure 8.

#### 3.9.1. Catalase

Catalase activity was evaluated in plasma and liver tissue homogenates in this investigation. Catalase activity was much lower in the plasma of TiO_2_-NPs-and-DMH-induced rats than in the plasma of control rats (*p* < 0.05). Catalase activity in TiO_2_-NPs-and-DMH-induced rats was recovered after apocynin therapy (Figure 8a and Table 9). Catalase activity was likewise reduced in TiO_2_-NPs-and-DMH-induced rats’ liver tissue homogenates when compared to control rats’ liver tissue homogenates; however, the differences between the two groups were not statistically significant (Figure 8a). 5-FU+CUR conjugate coated with pectin had an increased effect on catalase activity in TiO_2_-NPs-and-DMH-treated rats’ liver homogenates (Figure 8a). 

Detailed report were given in Figure 8a.

#### 3.9.2. Superoxide Dismutase (SOD)

Because of antioxidant enzymes such as superoxide dismutase (SOD) and catalase, cells have a well-organized defense system against oxidative-stress-mediated organelle damage and lipid peroxidation in biological membranes. Superoxide (O_2_) can be transformed by SOD to hydrogen peroxides (H_2_O_2_), which catalase can then convert to water. From the results obtained after evaluating the level of SOD in rat blood, after receiving their treatment, it was evident that group 1 showed an elevated concentration of SOD enzyme, along with groups with 3 and 4, compared to negative group 2. The results obtained for the treatment groups were in the same range as the positive control and showed good antioxidant activity. Reports regarding the comparison are given in Figure 8b and Table 9.

#### 3.9.3. Reduced Glutathione

When compared to the positive control, the cancer-induced negative control had a lower value. The reduced glutathione activity was larger in the presence of malignancy than in the control group. Following treatment, tests on the negative group revealed an increase in glutathione activity. The glutathione activity of distinct populations is seen in Figure 8c and Table 9.

#### 3.9.4. Malondialdehyde

Malondialdehyde is a parametric determination of lipid peroxidation, used for the evaluation of cellular damage created by the process of peroxidation, which was observed in the condition of cancer. The result was observed with a reduction in the concentration in the tested group compared with that of the negative control. The comparison is shown in Figure 8d and Table 9. 

### 3.10. Biochemical Estimation

Reports for the biochemical estimation of the different groups are given in Table 10.

#### 3.10.1. Liver Enzyme Analysis

**Figure 9:**
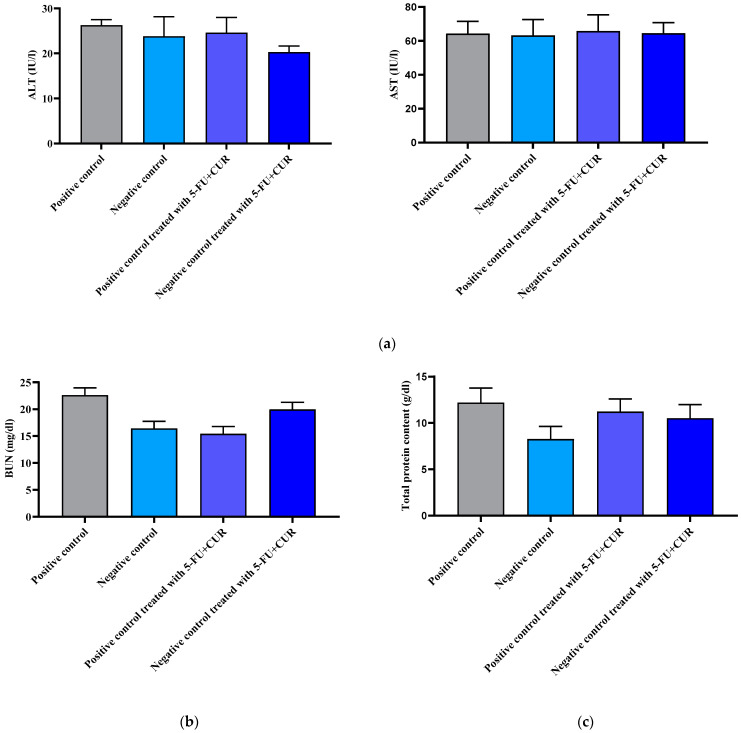
Biochemical estimation of different groups. (**a**) Liver enzyme analysis of different groups. (**b**) Kidney analysis report of different samples. (**c**) Total protein content estimation in different groups. The data are presented as the mean ± SD (*n* = 6 per group). The significance value (*p*-value) was found to be *p* < 0.001 (statistically significant). One-way ANOVA with a Bonferroni correction for multiple groups and two-tailed Student’s *t*-test for individual groups are shown, with no significant difference (*p* < 0.05) found.

Any injury to the liver or hepatic tissues appears to boost the activity of many marker enzymes, including ALT and AST. To evaluate the treatment progress specificity or its interference with other organs, liver enzyme analysis was carried out and is shown in Figure 9a. The values show that the liver function was not affected by the induction of cancer or by the treatment. Hence, it can be said that cancer metastasis did not actually begin and that the samples did not affect liver function (Figure 9a and Table 10).

#### 3.10.2. Kidney Analysis

For evaluating the effect of the treatment group on kidney function, the blood urea nitrogen indicator was measured and is shown in Figure 9b and Table 10. The values are coming into the normal range; hence, kidney function was not affected.

#### 3.10.3. Protein Content Analysis

Total protein content was estimated by using Lowry et al.’s method. A comparison is shown Figure 9c and Table 10. The values are coming into the normal range.

### 3.11. Histopathology Report

**Figure 10:**
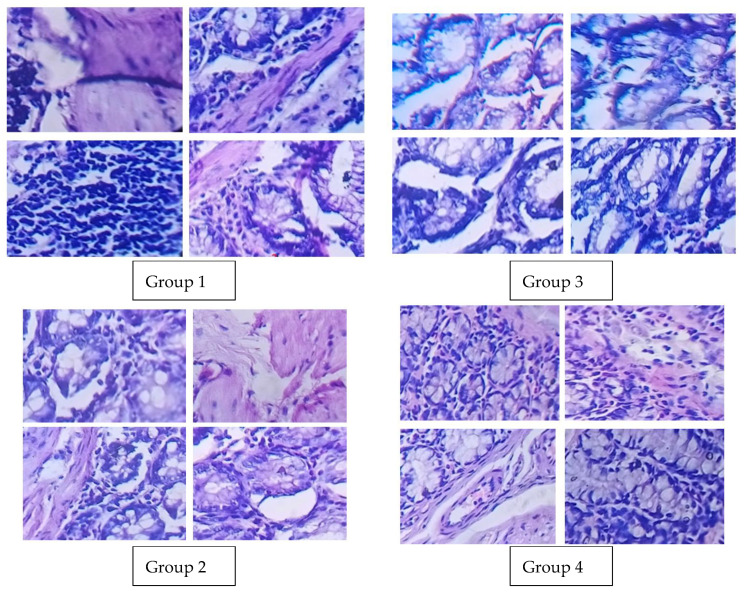
Histopathology images of different groups. Group 1: Positive control, Group 2: Negative control, Group 3: Positive control treated with 5-FU+CUR, Group 4: Negative control treated with 5-FU+CUR.

Using a 40× magnification microscope, images of stained samples were obtained on a scale of 0+, as shown in Figure 10. The histopathology reports of the different groups are given in Figure 10. Group 1 was the positive control, where the incidence of cancer was not reported, as the induction of cancer was not performed. In group 2, where the colorectal cancer was induced, the disruption of the cells and the cancer incidents can be observed. In group 3, the pectin-coated 5-FU and CUR was given as a treatment modality; this group showed no change or disruption of the cells, and the cell architecture was found to be normal. In group 4, where the negative control is given with the treatment modalities, the cancer incidents were reduced, and the cells returned to their normal architecture.

## 4. Discussion

Colorectal cancer is one of the most common kinds of cancer worldwide. As health-care systems have evolved, so has the number of cancer survivors [18]. These cancer survivors have several difficulties, including a significant chance of recurrence. Furthermore, because of therapy, a considerable proportion of patients may develop comorbidities. Concerns are being raised about the global growth in colorectal cancer incidence, as well as the emergence of medication resistance. Cancer therapy methods now available, like those for other malignancies, are either invasive or have major negative effects. As a result, better therapeutic alternatives are needed. 5-FU, an effective chemotherapy drug, is commonly used to treat colorectal cancer [32]. However, its toxicity to normal tissues and the development of cancer resistance are the primary impediments to successful cancer treatment, limiting its usage. CUR can enhance 5-FU-induced cytotoxicity, while decreasing negative side effects. CUR, derived from *Curcuma longa*, has piqued the interest of experts as a viable chemotherapy drug alternative. In small dosages, it is non-toxic and safe against colorectal cancer. It is worth noting that it has a special effect on colorectal cancer apoptotic effects. CUR’s utility in aquatic environments is limited because of its weak solubility and bioavailability [7].

A synergistic approach in the ratio of 1:4 works better, as has been proven in our previous research. 5-FU at 60 mg/kg body weight of the rat and curcumin at 240 mg/kg were given through an oral route. For the specific delivery to the colorectal region, pectin coating was done. Evidential support was also obtained using HCT-116 cell lines, as reported. The pH of the pectin-coated conjugate was in the range of 6–6.5. The bacteria and the microflora present in the colorectal region help in the specific delivery of the conjugate to the colorectal region by the degradation of the pectin-coated layer [33]. Furthermore, animal studies are conducted where blood and plasma analyses are carried out. In vitro cell line studies in HCT-116 cell lines and histopathology reports are also done for supporting evidence.

It was reported that there is an increase in the bodyweight of the animals with the induction of colorectal cancer using TiO_2_-NPS and DMH because of an increase in the blood sugar level. The level of TiO_2_-NPs in the blood plasma and colon were also noted: the reports show that the TiO_2_-NPs level was found to be high in the colon, which is the main reason for the inflammation in the colorectal region and the incidence of cancer [34]. With the hematological parameters’ evaluation, the RBC count and hemoglobin count were found to be decreased with cancer incidence, and subsequently, with treatment, the level came back to be normal. The WBC and platelet count were found to be higher than that of the positive control with the incidence of cancer and returned to normal with treatment with 5-FU and CUR conjugate coated with pectin. This is seen along with significant changes in the level of nitric oxide, advanced protein oxidation products, ROS, catalase, and superoxide dismutase, and reduced glutathione and malondialdehyde activity. The biochemical analysis shows no significant change in the level of induction and treatment. From this, we can conclude that the effect is not shown in the liver, kidney, or other organs or that metastasis did not occur.

## 5. Conclusions

Our study demonstrates that 5-FU CUR (60:240 mg/kg) conjugate coated with pectin has anticancer action in titanium dioxide + dimethylhydrazine-induced colorectal cancer in male rats. The powerful antioxidant and anti-inflammatory activities of 5-FU CUR (60:240 mg/kg) conjugate coated with pectin can be attributed to its anticancer activity, as several common markers of oxidative stress were down-regulated and antioxidant enzyme activities were upregulated in titanium dioxide+ dimethyl hydrazine treated male rats. More research is needed to determine the precise molecular mechanism of anti-cancer activity and its efficacy in a clinical setting of CRC illnesses.

## Figures and Tables

**Table 1:** Cytotoxicity comparison between 5-FU, CUR, 5-FU+CUR (1:4 ratio) coated with pectin, and 5-FU+CUR (1:4 ratio).

IC_50_ Value	48 h (µg/mL)	72 h (µg/mL)
5-FU	56.28 ± 1.2	48.24 ± 1.4
CUR	62.25 ± 1.5	55.25 ± 1.1
5-FU+CUR (1:4 ratio)	24.26 ± 0.8	20.26 ± 1.0
5-FU+CUR (1:4 ratio) coated with pectin	18.25 ± 1.2	12.26 ± 0.8

All the data are taken in triplicate, *n* = 3. The significance value (*p*-value) was found to be *p* < 0.05 (statistically significant).

**Table 2:** Bodyweight analysis during induction and treatment.

SL. No	Group	Initial Body Weight (g ± SD)	Final Body Weight- after Induction (10th Week) (g ± SD)	Final Body Weight- after Treatment (g ± SD)
1.	Positive control	236 ± 2.2	242 ± 1.6	250 ± 2.6
2.	Negative control	232 ± 2.5	316 ± 3.5	355 ± 2.4
3.	Positive control treated with 5-FU+CUR coated with pectin	224 ± 3.6	252 ± 2.5	260 ± 2.5
4.	Negative control treated with 5-FU+CUR coated with pectin	245 ± 1.6	345 ± 2.8	255 ± 3.8

The data are presented as the mean ± SD (*n* = 6 per group). The significance value (*p*-value) was found to be *p* < 0.0001 (statistically significant). One-way ANOVA with a Bonferroni correction for multiple groups and two-tailed Student’s *t*-test for individual groups are shown, with no significant difference (*p* < 0.05) found.

**Table 3:** The blood sugar levels of TiO_2_-and-DMH-induced rats were affected by 5-FU+CUR coated with pectin.

SL. No	Group	Blood Sugar Level mmol/L
1.	Positive control	5.65 ± 0.72
2.	Negative control	10.86 ± 1.28
3.	Positive control treated with 5-FU+CUR with pectin coating	6.26 ± 1.18
4.	Negative control treated with 5-FU+CUR with pectin coating	9.25 ± 2.16

The data are presented as the mean ± SD (*n* = 6 per group). The significance value (*p*-value) was found to be *p* < 0.05 (statistically significant).

**Table 4:** The level of TiO_2_-NPs in the blood and colorectal region.

SL. No	Group	TiO_2_ Level in Blood Plasma (µg/mL)	TiO_2_ Level in the Colon (µg/g)
1.	Positive control	0.00 ± 0.00	0.00 ± 0.00
2.	Negative control	3.22 ± 0.25	52.15 ± 1.22
3.	Positive control treated with 5-FU+CUR with pectin coating	0.00 ± 0.00	0.00 ± 0.00
4.	Negative control treated with 5-FU+CUR with pectin coating	0.26 ± 0.12	3.23 ± 1.23

The data are presented as the mean ± SD (*n* = 6 per group). The significance value (*p*-value) was found to be *p* < 0.0001 (statistically significant).

**Table 5:** Hematological parameters testing report.

SL. No	Group	RBC Content (10^6^/mm^3^ ± SD)	WBC Content (10^3^/mm^3^ ± SD)	Hemoglobin Content (g/dL ±SD)	Platelet Content (10^4^/mm^3^ ± SD)
1.	Positive control	8.35 ± 2.55	8.25 ± 2.35	14.30 ± 1.50	25.10 ± 2.90
2.	Negative control	5.55 ± 1.20	17.80 ± 2.35	4.12 ± 1.25	39.44 ± 3.22
3.	Positive control treated with 5-FU+CUR with pectin coating	8.25 ± 1.22	8.25 ± 2.24	14.25 ± 2.16	25.22 ± 1.22
4.	Negative control treated with 5-FU+CUR with pectin coating	7.89 ± 1.05	9.75 ± 1.56	13.25 ± 2.25	26.26 ± 2.22

The data are presented as the mean ± SD (*n* = 6 per group). The significance value (*p*-value) was found to be *p* < 0.0001 (statistically significant). The R^2^ Value was found to be 0.8851 (one-way ANOVA with a Bonferroni correction for multiple groups and two-tailed Student’s *t*-test for individual groups). Bartlett’s statistic correlation was found to be 6.154 with a *p*-value of 0.1044; no significant difference (*p* < 0.05) was found.

**Table 6:** Effect of nitric oxide.

SL. No	Group	Nitric Oxide nmol/gm Tissue
1.	Positive control	13.65 ± 0.72
2.	Negative control	32.86 ± 1.28
3.	Positive control treated with 5-FU+CUR with pectin coating	14.26 ± 1.18
4.	Negative control treated with 5-FU+CUR with pectin coating	17.0 ± 2.16

The data are presented as the mean ± SD (*n* = 6 per group). The significance value (*p*-value) was found to be *p* < 0.05 (statistically significant).

**Table 7:** Effect of 5-FU CUR conjugate on advanced protein oxidation products (APOP).

SL. No	Group	Group	Advanced Protein Oxidation Products (APOP) nmol/mg Protein
1.	Positive control	Control	396.60 ± 1.72
2.	Negative control	Diseased	837.86 ± 3.48
3.	Positive control was treated with 5-FU+CUR coated with pectin.	Control+ treated	406.26 ± 1.38
4.	Negative control treated with 5-FU+CUR with pectin coating	Diseased+ treated	428.0 ± 2.26

The data are presented as the mean ± SD (*n* = 6 per group). The data are presented as the mean ± SD (*n* = 6 per group). The significance value (*p*-value) was found to be *p* < 0.05 (statistically significant).

**Table 8:** Effect of 5-FU CUR conjugate.

SL. No	Group	ROS-Fluorescence (FC)
1	Positive control	1000 ± 20
2	Negative control	1500 ± 25
3	Positive control treated with 5-FU+CUR with pectin coating	900 ± 22
4	Negative control treated with 5-FU+CUR with pectin coating	1100 ± 17

The data are presented as the mean ± SD (*n* = 6 per group). The significance value (*p*-value) was found to be *p* < 0.05 (statistically significant).

**Table 9:** Cumulative antioxidant estimation.

SL. No	Group	Catalase (µmol/min/mg Protein)	Superoxide Dismutase (U/mL)	Reduced Glutathione (nmol/mg Protein)	Malondialdehyde (TBAR/mg Protein)
1.	Positive control	0.69 ± 0.02	125.23 ± 11.26	1.22 ± 0.02	24.70 ± 8.72
2.	Negative control	0.25 ± 0.03	42.73 ± 10.23	0.46 ± 0.02	65.76 ± 12.38
3.	Positive control treated with 5-FU+CUR with pectin coating	0.62 ± 0.01	115.26 ± 9.26	1.24 ± 0.04	26.26 ± 7.28
4.	Negative control treated with 5-FU+CUR with pectin coating	0.52 ± 0.07	98.22 ± 11.02	0.96 ± 0.01	30.50 ± 8.26

The data are presented as the mean ± SD (*n* = 6 per group). The significance value (*p*-value) was found to be *p* < 0.001 (statistically significant). One-way ANOVA with a Bonferroni correction for multiple groups and two-tailed Student’s *t*-test for individual groups are shown, with no significant difference (*p* < 0.05) found.

**Table 10:** Biochemical estimation.

SL. No	Group	Liver Enzymes		Kidney Analysis	Protein Content Analysis
		ALT (IU/L)	AST (IU/L)	BUN (mg/dL)	Total Protein Content (g/dL)
1.	Positive control	26.25 ± 1.26	64.30 ± 7.25	22.62 ± 1.35	12.21 ± 1.56
2.	Negative control	23.82 ± 4.32	63.26 ± 9.32	16.42 ± 1.32	8.27 ± 1.36
3.	Positive control treated with 5-FU+CUR with pectin coating	24.62 ± 3.36	65.86 ± 9.56	15.43 ± 1.36	11.23 ± 1.36
4.	Negative control treated with 5-FU+CUR with pectin coating	20.28 ± 1.38	64.52 ± 6.32	19.96 ± 1.33	10.52 ± 1.46

The data are presented as the mean ± SD (*n* = 6 per group). The significance value (*p*-value) was found to be *p* < 0.001 (statistically significant). One-way ANOVA with a Bonferroni correction for multiple groups and two-tailed Student’s *t*-test for individual groups are shown, with no significant difference (*p* < 0.05) found.

## Data Availability

The data presented in this study are available on request from the corresponding author.
